# Leveraging human genetic data to investigate the cardiometabolic effects of glucose-dependent insulinotropic polypeptide signalling

**DOI:** 10.1007/s00125-021-05564-7

**Published:** 2021-09-09

**Authors:** Ville Karhunen, Iyas Daghlas, Verena Zuber, Marijana Vujkovic, Anette K. Olsen, Lotte Bjerre Knudsen, William G. Haynes, Joanna M. M. Howson, Dipender Gill

**Affiliations:** 1 Department of Epidemiology and Biostatistics, Imperial College London, London, UK; 2 Research Unit of Mathematical Sciences, University of Oulu, Oulu, Finland; 3 Center for Life Course Health Research, University of Oulu, Oulu, Finland; 4 Harvard Medical School, Boston, MA USA; 5 Medical Research Council Biostatistics Unit, Cambridge Institute of Public Health, Cambridge, UK; 6 Corporal Michael J. Crescenz VA Medical Center, Philadelphia, PA USA; 7 Department of Medicine, University of Pennsylvania Perelman School of Medicine, Philadelphia, PA USA; 8 Global Drug Discovery, Novo Nordisk A/S, Måløv, Denmark; 9 Novo Nordisk Research Centre Oxford, Old Road Campus, Oxford, UK; 10 Radcliffe Department of Medicine, University of Oxford, Oxford, UK; 11 Clinical Pharmacology Group, Pharmacy and Medicines Directorate, St George’s University Hospitals NHS Foundation Trust, London, UK; 12 Clinical Pharmacology and Therapeutics Section, Institute for Infection and Immunity, St George’s, University of London, London, UK

**Keywords:** Cardiometabolic disease, Co-localisation, Glucose-dependent insulinotropic polypeptide, Mendelian randomisation, Type 2 diabetes mellitus

## Abstract

**Aims/hypothesis:**

The aim of this study was to leverage human genetic data to investigate the cardiometabolic effects of glucose-dependent insulinotropic polypeptide (GIP) signalling.

**Methods:**

Data were obtained from summary statistics of large-scale genome-wide association studies. We examined whether genetic associations for type 2 diabetes liability in the *GIP* and *GIPR* genes co-localised with genetic associations for 11 cardiometabolic outcomes. For those outcomes that showed evidence of co-localisation (posterior probability >0.8), we performed Mendelian randomisation analyses to estimate the association of genetically proxied GIP signalling with risk of cardiometabolic outcomes, and to test whether this exceeded the estimate observed when considering type 2 diabetes liability variants from other regions of the genome.

**Results:**

Evidence of co-localisation with genetic associations of type 2 diabetes liability at both the *GIP* and *GIPR* genes was observed for five outcomes. Mendelian randomisation analyses provided evidence for associations of lower genetically proxied type 2 diabetes liability at the *GIP* and *GIPR* genes with lower BMI (estimate in SD units −0.16, 95% CI −0.30, −0.02), C-reactive protein (−0.13, 95% CI −0.19, −0.08) and triacylglycerol levels (−0.17, 95% CI −0.22, −0.12), and higher HDL-cholesterol levels (0.19, 95% CI 0.14, 0.25). For all of these outcomes, the estimates were greater in magnitude than those observed when considering type 2 diabetes liability variants from other regions of the genome.

**Conclusions/interpretation:**

This study provides genetic evidence to support a beneficial role of sustained GIP signalling on cardiometabolic health greater than that expected from improved glycaemic control alone. Further clinical investigation is warranted.

**Data availability:**

All data used in this study are publicly available. The scripts for the analysis are available at: https://github.com/vkarhune/GeneticallyProxiedGIP.

**Graphical abstract:**

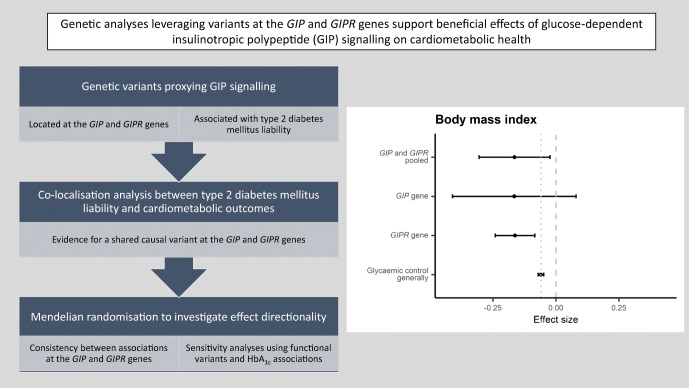

**Supplementary Information:**

The online version contains peer-reviewed but unedited supplementary material available at 10.1007/s00125-021-05564-7.

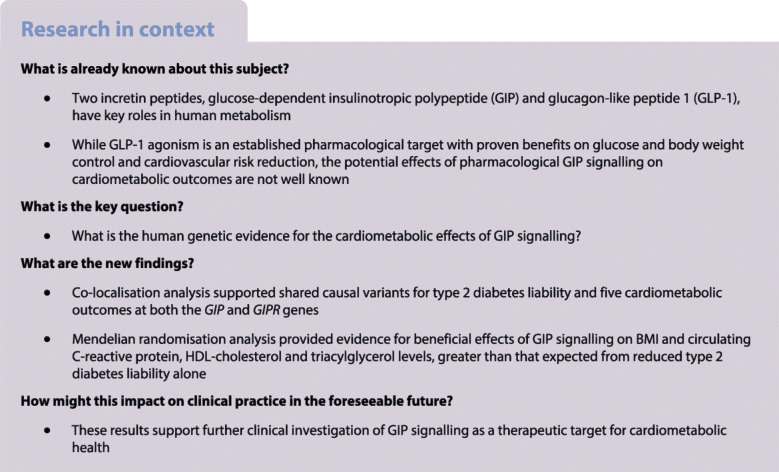



## Introduction

Glucose-dependent insulinotropic polypeptide (or gastric inhibitory polypeptide, GIP) is an incretin peptide that stimulates insulin secretion after oral nutrient intake. Both GIP and glucagon-like peptide 1 (GLP-1) are involved in regulating energy homeostasis [1]. GLP-1 agonism is an established pharmacological target for treating type 2 diabetes and obesity, however it is unclear whether pharmacological GIP agonism represents a similar therapeutic opportunity [2]. Here, we leverage human genetic data to investigate the potential of targeting GIP signalling for the treatment of cardiometabolic disease.

## Methods

### Overall study design

We investigated whether genetic associations for type 2 diabetes liability co-localised with genetic associations for 11 cardiometabolic outcomes (Table 1) at the *GIP* and *GIPR* genes. For those outcomes that showed evidence for co-localisation, we performed Mendelian randomisation (MR) analyses to investigate the association of genetically proxied glucose-dependent insulinotropic polypeptide (GIP) signalling with the cardiometabolic outcomes, and whether these estimates are greater than that expected from reduced type 2 diabetes liability alone. Further details are given in the electronic supplementary material (ESM) Methods.

**Table 1:** Genome-wide association studies used to obtain the summary statistics

Phenotype	Sample size	Cases	Controls	Source^a^
Exposures				
Type 2 diabetes^b^		74,124	824,006	Mahajan et al, 2018
Type 2 diabetes^c^		228,499	1,178,783	Vujkovic et al, 2020
HbA_1c_	344,182			Neale lab 2020^d^
Outcomes				
Disease outcomes				
Chronic kidney disease		64,164	561,055	Wuttke et al, 2019
Coronary artery disease		60,801	123,504	Nikpay et al, 2015
HF		47,309	930,014	Shah et al, 2020
Ischaemic stroke		34,217	406,111	Malik et al, 2018
Cardiometabolic traits				
Alanine aminotransferase	344,136			Neale lab 2020
BMI	484,680			Pulit et al. 2018
CRP	343,524			Neale lab 2020
Systolic BP	745,820			Evangelou et al. 2018
Lipids				
HDL-C	315,133			Neale lab 2020
LDL-C	343,621			Neale lab 2020
Triacylglycerol	343,992			Neale lab 2020

^a^The references for the original studies are given in the ESM

^b^Used for co-localisation analysis

^c^Used for MR analysis

^d^All Neale lab 2020 GWAS summary statistics are available at: http://www.nealelab.is/uk-biobank

LDL-C, LDL-cholesterol

### Genetic association estimates

Genetic association estimates for SNPs with type 2 diabetes liability, HbA_1c_ levels and the considered cardiometabolic outcomes were obtained from genome-wide association study (GWAS) summary statistics (Table 1). The individual studies had previously obtained relevant ethical approval and participant consent.

### Statistical analyses

We used co-localisation analysis to compare the genetic association signals for type 2 diabetes liability and each cardiometabolic outcome for variants within *GIP* and *GIPR*. The ‘coloc’ method applied here examines the likelihood of a shared causal variant for both exposure and outcome [3]. Co-localisation was declared if the posterior probability (PP) for a model with a shared causal variant exceeded 0.8 (ESM Methods). For the outcomes where co-localisation analysis suggested separate causal variants for type 2 diabetes liability and the outcome, co-localisation was re-run after excluding the variants that were in linkage disequilibrium (*r*^*2*^ > 0.2) with the most likely causal SNP for the outcome (ESM Methods).

The outcomes that showed evidence for co-localisation were taken forward for MR analysis. In MR, genetic variants are used as proxies for an exposure (here, GIP signalling) to examine its potential causal effect on an outcome, and the method can be applied to investigate drug effects [4]. Given the known role of GIP signalling on improving glycaemic control in healthy individuals [5], we identified genetic proxies as SNPs located within *GIP* and *GIPR* genes that associated with type 2 diabetes liability at *p* < 5 × 10^−6^ and also associated with HbA_1c_ levels at *p* < 0.05 with a concordant direction, and applied clumping by excluding variants with *r*^*2*^ > 0.1 with the lead SNP. Prior filtering of genetic variants was applied based on the co-localisation analysis, so that variants exhibiting potential horizontal pleiotropy were removed (ESM Methods).

The main MR analysis was conducted by pooling the associations of all proxy variants from both the *GIP* and *GIPR* genes using the random-effects inverse-variance weighted method. To compare the associations of genetically proxied GIP signalling with improved glycaemic control more generally, we compared the main MR results to that of a general reduction in type 2 diabetes liability and improved glycaemic control using variants across the genome that associated with type 2 diabetes liability at *p* < 5 × 10^−6^ and HbA_1c_ levels at *p* < 0.05 with a concordant direction, excluding variants within *GIP* and *GIPR* (ESM Methods). In sensitivity analysis, we performed MR using exposure genetic association estimates for HbA_1c_ levels, rather than type 2 diabetes liability (ESM Methods). As a final sensitivity analysis, we performed MR using functionally relevant variants or expression quantitative trait loci (eQTL, ESM Methods).

## Results

Co-localisation analysis showed evidence of a shared causal variant for type 2 diabetes liability and eight outcomes at the *GIP* gene, and six outcomes at the *GIPR* gene (PP > 0.8, ESM Table 1). For the five outcomes of heart failure (HF), BMI, C-reactive protein (CRP) levels, HDL-cholesterol (HDL-C) and triacylglycerols, there was evidence for Co-localisation also protects at both *GIP* and *GIPR*.

**Fig. 1:**
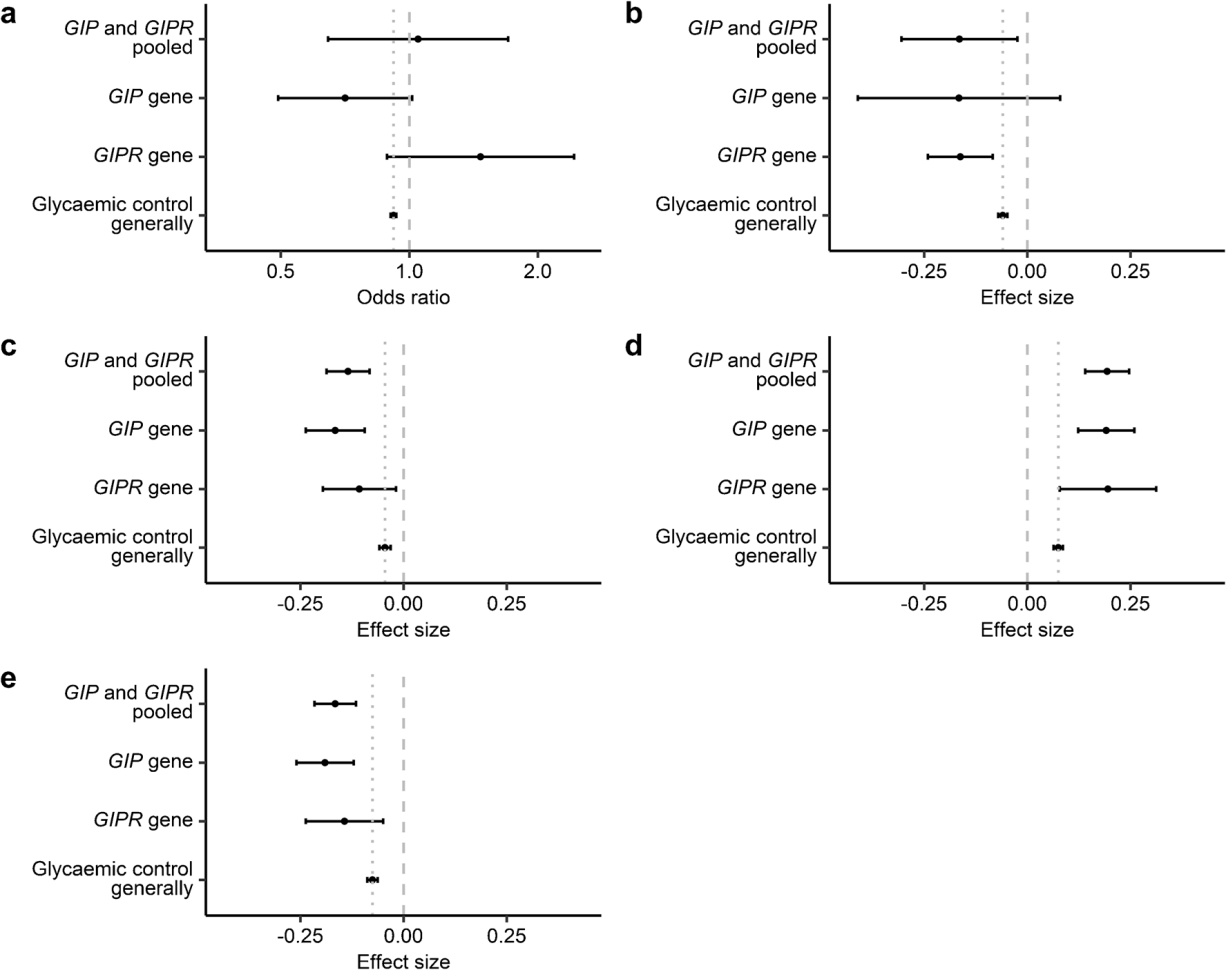
(**a**) ORs for risk of HF and (**b–e**) effect size estimates (MR β coefficients for BMI [**b**], CRP [**c**], HDL-C [**d**] and triacylglycerol levels [**e**], all in SD units) and their 95% CIs per halving the odds of genetically proxied type 2 diabetes liability. The dashed vertical line represents the null, and the dotted vertical line represents the estimates for glycaemic control generally

The co-localising outcomes were taken forward to MR analysis, where increased genetically proxied GIP signalling (using variants given in ESM Tables 2–4) was associated with lower BMI (estimate and its 95% CI in SD units per halving the genetically proxied odds of type 2 diabetes = −0.16 [−0.30, −0.02]), CRP levels (−0.13 [−0.19, −0.08]) and triacylglycerol levels (−0.17 [−0.22, −0.12]), and higher HDL-C levels (0.19 [0.14, 0.25]; Fig. 1; ESM Table 4). For these outcomes, the MR estimates were similar when using variants from *GIP* and *GIPR* genes separately (Fig. 1; ESM Table 4). The MR estimate for risk of HF was inconclusive (OR per halving the odds of type 2 diabetes [95% CI] = 1.05 [0.65, 1.70]; Fig. 1; ESM Table 4). There was evidence of a larger association of genetically proxied GIP signalling compared with genetically proxied reduced type 2 diabetes liability more generally for CRP, HDL-C and triacylglycerol levels (all *p* < 0.001), but not strongly for BMI (*p* = 0.07, ESM Table 4). Similar results were obtained when using variant–exposure associations for HbA_1c_ levels rather than for type 2 diabetes liability (Pearson correlation of the MR β estimates = 0.99; ESM Table 5; ESM Figs 1 and 2), and when using missense variant rs2291725 in *GIP* or eQTL variant rs12709891 in *GIPR* (ESM Tables 2 and 6; ESM Fig. 3).

For those outcomes that co-localised only at one genomic locus, there was evidence for association between genetically proxied GIP signalling and lower risk of coronary artery disease (OR [95% CI] = 0.51 [0.37, 0.71]), lower alanine aminotransferase (−0.13 [−0.20, −0.07]) and lower systolic BP (−0.18 [−0.25, −0.12]) at the *GIP* locus, with all these the estimates exceeding those obtained for reduced type 2 diabetes liability more generally (ESM Fig. 4; ESM Table 7).

## Discussion

Our genetic analyses using human data provide consistent support for favourable effects of sustained GIP signalling on BMI, CRP, HDL-C and triacylglycerol levels. The MR estimates for CRP, HDL-C and triacylglycerol levels exceeded and were statistically heterogeneous to those obtained for reduced type 2 diabetes liability more generally, suggesting additional mechanisms specific to GIP signalling. The MR results were replicated in analyses restricted to functionally relevant variants.

Although a dual GIP and GLP-1 receptor agonist has shown efficacy for glucose control and weight loss in clinical trials of patients with type 2 diabetes [6, 7], it is not clear how much of the observed effect is specifically attributable to GIP agonism. Preclinical studies have supported that sustained GIP receptor (GIPR) agonism prevents weight gain and enhances weight loss in diet-induced obese mice [8, 9]. Our analyses using human genetic data provide further complementary evidence of the beneficial cardiometabolic effects of sustained GIP signalling.

We observed discrepancy in the MR estimates for HF risk that were generated when considering the *GIP* gene (lower risk) as compared with the *GIPR* gene (higher risk). Although a cardiovascular outcomes trial of dipeptidyl peptidase 4 inhibition with saxagliptin found increased hospitalisation rates for HF [10], this was not found for sitagliptin [11]. Further work is required to ascertain whether our findings offer any mechanistic or clinical insight in this regard.

The use of randomly allocated genetic variants to proxy drug effects in the MR paradigm is more robust to environmental confounding that can hinder causal inference in observational studies [4]. We selected the genetic proxies for sustained GIP signalling based on its known biological effects in healthy individuals, namely improved glycaemic control and reduced liability to type 2 diabetes [5]. Previous work selected a variant to proxy GIP signalling based on its relation to fasting GIP levels, and in contrast to our current findings produced MR results to support a detrimental effect of GIP signalling on coronary artery disease risk [12]. Further work is required to clarify how different genetic variants at the *GIPR* gene might relate to GIP signalling and its consequent downstream metabolic effects.

Our work has limitations. The genetic associations were obtained from GWAS on mainly European ancestry individuals, and these results may not generalise to other ancestries. The findings do not extend to effects of other glucose-lowering medications that indirectly alter GIP signalling, and may not be applicable to individuals with diabetes, in whom the physiological effects of GIP signalling may be altered [13]. Finally, the genetic variants employed as instruments proxy the effect of lifelong alterations in GIP signalling and therefore the MR results should not be directly extrapolated to quantitatively estimate the clinical effect of short-term GIPR agonism [4]. Of relevance, recent evidence has supported that long-term GIPR agonism desensitises adipocyte GIPR activity in a manner resembling acute GIPR antagonism [8].

In conclusion, by leveraging human genetic data, we provide evidence of favourable effects of sustained GIP signalling on BMI, CRP, HDL-C and triacylglycerol levels. These results support further clinical investigation of GIP agonism as a therapeutic target for cardiometabolic disease.

## Supplementary Information

ESM (PDF 619 kb)

## Abbreviations

CRP: C-reactive protein
eQTL: Expression quantitative trait loci
GIP: Glucose-dependent insulinotropic polypeptide
GIPR: Glucose-dependent insulinotropic polypeptide receptor
GLP-1: Glucagon-like peptide 1
GWAS: Genome-wide association study
HDL-C: HDL-cholesterol
HF: Heart failure
MR: Mendelian randomisation
PP: Posterior probability
