# Supplementary material for: Leveraging human genetic data to investigate the cardiometabolic effects of glucose-dependent insulinotropic polypeptide signalling
Source: Diabetologia. 2021 Sep 9;64(12):2773–8. doi: 10.1007/s00125-021-05564-7 (PMC8563538; doi:10.1007/s00125-021-05564-7)
Supplement: Supplementary file 1 — (PDF 619 kb) [file 125_2021_5564_MOESM1_ESM.pdf]

Leveraging human genetic data to investigate the cardiometabolic effects of glucose-dependent insulinotropic polypeptide signaling: Electronic Supplementary Material (ESM)

Corresponding authors: Ville Karhunen (ORCID: 0000-0001-6064-1588) and Dipender Gill (ORCID: 0000-0001-7312-7078)

Table of Contents

ESM Methods....2
ESM Table 1....7
ESM Table 2....8
ESM Table 3....9
ESM Table 4....10
ESM Table 5....11
ESM Table 6....12
ESM Table 7....13
ESM Fig. 1....14
ESM Fig. 2....15
ESM Fig. 3....16
ESM Fig. 4....17
References....18

ESM Methods

Genetic association estimates

The genetic association estimates were obtained from large genome-wide association studies (GWAS) [1–9]. We utilised the known effect of glucose-dependent insulinotropic polypeptide (GIP) on improving glycemic control in healthy individuals to proxy GIP signaling [10]. At the *GIP* (hg19 build: chromosome 17, position 47,035,916–47,045,958) and *GIPR* (chromosome 19, position 46,171,502–46,186,982) genes, single-nucleotide polymorphism (SNP) associations with type 2 diabetes liability were used as the main exposure of interest, preferred over genetic associations with HbA_{1c} levels in the main analysis due to the larger GWAS sample size. For Mendelian randomisation (MR), the genetic associations with type 2 diabetes were complemented by also using the genetic associations with HbA_{1c} levels, as described below. Palindromic SNPs with minor allele frequency > 0.4 were excluded. Only SNPs with summary statistics available for both traits of each exposure–outcome pair were used.

In co-localisation, genetic associations for type 2 diabetes liability were obtained from the analysis of European-ancestry individuals performed by Mahajan et al., as summary statistics for all variants are publicly available [1]. In MR, trans-ancestry type 2 diabetes liability genetic associations for the variants modelled as instruments were obtained from Vujkovic et al., and were used due to the larger GWAS sample size [2]. Association estimates for the lead variants were similar between these two studies.

We selected the following eleven cardiometabolic traits as outcomes: coronary artery disease, chronic kidney disease, heart failure, ischemic stroke, alanine aminotransferase, body

mass index, C-reactive protein, high-density lipoprotein cholesterol, low-density lipoprotein cholesterol, systolic blood pressure, and triacylglycerol (triglycerides). The trait definitions for the exposures and outcomes are given in their original publications [3–9].

Statistical analysis

Co-localisation. To examine whether exposure (type 2 diabetes liability) shares a common causal variant with each outcome within *GIP* and *GIPR*, we conducted co-localisation analysis using coloc [11], a Bayesian method which calculates posterior probabilities (PP) for the following competing models:

Model 1: The genomic region contains a variant influencing only the exposure.

Model 2: The genomic region contains a variant influencing only the outcome.

Model 3: The genomic region contains two separate variants, one influencing the exposure and the other influencing the outcome.

Model 4: The genomic region contains a variant influencing both exposure and outcome.

Co-localisation of exposure and outcome within the gene is declared if PP > 0.8 for Model 4, and these traits were taken forward to MR (described below).

A high PP for Model 3 supports separate causal variants for the exposure and the outcome traits and implies horizontal pleiotropy, where the variants affect the two traits via separate causal pathways. In such a scenario (PP > 0.8 for Model 3), variants that were in linkage disequilibrium (LD, $r^2 > 0.2$ based on the European reference population of 1000 Genomes Project) with the most likely causal SNP for the outcome (the variant with highest PP under Model 2) were

excluded, and co-localisation was repeated for the remaining SNPs. This filtering aims to remove variants that exhibit horizontal pleiotropy: if there are variants within *GIP* or *GIPR* that are associated with the outcome, but not type 2 diabetes liability, it is likely that these variant-outcome associations are not due to GIP signaling, and therefore not relevant for analysing in relation to this signaling pathway. Co-localisation also protects against potential genetic confounding from LD in MR: if the traits have separate causal variants within the same genomic locus and these variants are in LD with each other, MR may provide evidence for association, even when the detected association is caused purely by the LD between the variants.

Mendelian randomisation. For those outcomes that showed evidence of co-localisation with type 2 diabetes liability in either *GIP* and *GIPR*, we conducted two-sample MR using summary data [12] to further investigate causal effects of GIP signaling on the considered outcomes. To proxy the effect of GIP signaling, we selected SNPs located in the *GIP* and *GIPR* genes that associated with type 2 diabetes liability at $p < 5\times10^{-6}$ and also associated with HbA_{1c} levels at $p < 0.05$ with a concordant direction. This *p*-value threshold for type 2 diabetes liability was used to allow for inclusion of more instrumental variables that marginally did not reach the conventional *p*-value threshold of $p < 5\times10^{-8}$. *F* statistics were estimated as a measure of instrument strength, based on the variance explained calculated by assuming a logistic distribution for type 2 diabetes liability [13]. The selected variants were clumped so that variants with $r^2 > 0.1$ with the lead SNP were excluded. If co-localisation analysis suggested two separate causal variants (PP > 0.8 for Model 3), variants that were in LD ($r^2 > 0.2$) with the most likely causal SNP on the outcome (the variant with highest PP under Model 2) were excluded as above, and only the remaining variants were used for MR.

MR analyses were conducted using the random-effects inverse-variance weighted method, where proxy SNP variant–outcome association estimates (given on the log-odds scale for binary outcomes, or in standard deviation units for continuous outcomes) are regressed on variant–exposure association estimates (given on the log-odds scale for type 2 diabetes liability), weighted by the precision of variant–outcome estimates and the intercept constrained to zero. The main MR analysis combines all proxy variants from *GIP* and *GIPR* genes. We also report the MR results separately for *GIP* variants and *GIPR* variants. The Wald ratio was used as the MR estimate if only one SNP was available. The main MR estimates are reported per halving the odds of genetic liability to type 2 diabetes.

To compare the results of GIP signaling to reduced type 2 diabetes liability more generally, we compared the MR estimate to that obtained when using variants related to reduced type 2 diabetes liability (and improved glycaemic control) from throughout the genome, excluding variants within the *GIP* and *GIPR* genes. Similar criteria for instrumental variables were used for the genome-wide selection of variants, i.e. uncorrelated ($r^2 < 0.01$) variants that associated with type 2 diabetes liability at $p < 5\times10^{-6}$ and also associated with HbA_{1c} levels at $p < 0.05$ with a concordant direction. The standard error of the difference between the main MR and the genome-wide MR results was estimated using the propagation of error method. As we were interested in whether the absolute value of the pooled *GIP* and *GIPR* MR estimate is larger than the MR estimate for type 2 diabetes liability more generally, a one-sided *p*-value was reported.

As a sensitivity analysis, the same MR analyses were run using HbA_{1c} genetic association estimates, instead of liability to type 2 diabetes. Pearson correlation coefficient was used to compare the log-odds of type 2 diabetes MR estimates with HbA_{1c} MR estimates. Finally, we

performed the MR analyses restricting to common variants (minor allele frequency > 0.01) that associated with type 2 diabetes liability at $p < 5\times10^{-6}$, did not show evidence for pleiotropic effects in co-localisation and were either (i) of functional relevance (loss-of-function, missense or nonsense variants, or deletions), or (ii) proxies identified as in the previous section that were also expression quantitative trait loci (eQTL). The search was conducted via PhenoScanner database version 2 [14, 15], and using GTEx database version 8 for eQTL associations [16]. Variants were considered eQTL if they associated with expression of the gene in which they are located at $p < 5\times10^{-8}$ in fixed-effects meta-analysis across all tissues.

Measuring the strength of evidence. We interpret the evidence based on the estimates, the widths of the confidence intervals, and the consistency of the MR results obtained when using type 2 diabetes liability and HbA_{1c} as exposures. No explicit p-value threshold was applied for statistical significance [17].

ESM Table 1. Co-localisation analysis results.

	***GIP* gene**		***GIPR* gene**	
**Outcome**	**PP**	**Variants excluded**[a]	**PP**	**Variants excluded**[a]
Disease outcomes				
Chronic kidney disease	0.27		0.04	
Coronary artery disease	>0.99		0.09	
Heart failure	0.89		0.85	
Ischemic stroke	0.08		0.35	
Cardiometabolic traits				
Alanine aminotransferase	0.89		0.63	
Body mass index	>0.99		>0.99	rs1800437
C-reactive protein	0.98		0.95	
Systolic blood pressure	>0.99		0.02	rs11672660
Lipids				
High-density lipoprotein cholesterol	0.92		>0.99	
Low-density lipoprotein cholesterol	0.03	rs75421129	>0.99	
Triacylglycerol	0.88		>0.99	

PP = posterior probability for the model with a shared variant influencing both exposure and outcome. [a]In the case of evidence for a separate causal variant for the outcome, this variant and any other correlated variants ($r^2 > 0.2$) were excluded and colocalization analysis was repeated.

ESM Table 2. Genetic variants used to proxy GIP signaling in Mendelian randomisation analyses, and their association estimates with type 2 diabetes liability (log-odds scale) and HbA_{1c} levels (per mmol/mol). CHR: chromosome; POS: position; EA: effect allele; NEA: non-effect allele; EAF: effect allele frequency; SE: standard error; F: F-statistic.

							**Type 2 diabetes liability**			**HbA_{1c}**	
**rsID**	**CHR**	**POS**	**Gene**	**EA**	**NEA**	**EAF**	**Beta (SE)**	**p**	***F*[a]**	**Beta (SE)**	**p**
rs55936433	17	47036042	*GIP*	C	A	0.73	0.042 (0.004)	2.2×10^{-22}	161	0.072 (0.017)	1.5×10^{-4}
rs58274617	17	47036409	*GIP*	C	G	0.38	0.037 (0.007)	5.0×10^{-8}	147	0.073 (0.022)	2.0×10^{-4}
rs2291725[b]	17	47039132	*GIP*	C	T	0.54	0.034 (0.004)	4.3×10^{-8}	135	0.081 (0.015)	1.2×10^{-6}
rs9749185	19	46175416	*GIPR*	A	G	0.28	0.062 (0.005)	1.2×10^{-31}	362	0.159 (0.026)	2.7×10^{-11}
rs55669001[c]	19	46177235	*GIPR*	C	T	0.27	0.061 (0.005)	2.7×10^{-31}	344	0.161 (0.026)	1.2×10^{-11}
rs12709891[d]	19	46185217	*GIPR*	A	C	0.31	0.036 (0.004)	6.9×10^{-18}	130	0.050 (0.018)	4.6×10^{-5}

[a]Calculated by assuming a logistic distribution for the liability. [b]Missense variant, only used in sensitivity analysis. [c]Variant in high linkage disequilibrium (r^2=0.99) with rs9749185; used only if rs9749185 not available in outcome summary statistics. [d]Expression quantitative trait locus for *GIPR* (p=7×10^{-21} for *GIPR* expression in the meta-analysis across all tissues).

ESM Table 3. Genetic associations of variants used to proxy GIP signaling in Mendelian randomisation analysis for each outcome. EA: effect allele; NEA: non-effect allele; SE: standard error.

Outcome	rsID	Gene	EA	NEA	Beta	SE
Heart failure	rs55936433	*GIP*	C	A	0.028	0.009
	rs58274617	*GIP*	C	G	0.004	0.012
	rs2291725[a]	*GIP*	C	T	0.020	0.008
	rs55669001	*GIPR*	C	T	-0.052	0.013
	rs12709891	*GIPR*	A	C	-0.003	0.009
Body mass index	rs55936433	*GIP*	C	A	0.015	0.002
	rs58274617	*GIP*	C	G	-0.001	0.003
	rs2291725[a]	*GIP*	C	T	0.007	0.002
	rs12709891	*GIPR*	A	C	0.009	0.002
C-reactive protein	rs55936433	*GIP*	C	A	0.011	0.003
	rs58274617	*GIP*	C	G	0.007	0.003
	rs2291725[a]	*GIP*	C	T	0.005	0.002
	rs9749185	*GIPR*	A	G	0.006	0.004
	rs12709891	*GIPR*	A	C	0.008	0.003
High-density lipoprotein cholesterol	rs55936433	*GIP*	A	G	-0.011	0.003
	rs58274617	*GIP*	C	G	-0.011	0.003
	rs2291725[a]	*GIP*	C	T	-0.012	0.002
	rs55669001	*GIPR*	C	T	-0.013	0.005
	rs12709891	*GIPR*	A	C	-0.014	0.003
Triacylglycerol	rs55936433	*GIP*	A	G	0.011	0.003
	rs58274617	*GIP*	C	G	0.012	0.003
	rs2291725[a]	*GIP*	C	T	0.014	0.002
	rs55669001	*GIPR*	C	T	0.009	0.004
	rs12709891	*GIPR*	A	C	0.010	0.003
Coronary artery disease[b]	rs55936433	*GIP*	C	A	0.048	0.010
	rs58274617	*GIP*	C	G	0.023	0.013
Alanine aminotransferase[b]	rs55936433	*GIP*	C	A	0.007	0.002
	rs58274617	*GIP*	C	G	0.009	0.003
Low-density lipoprotein cholesterol[c]	rs55669001	*GIPR*	C	T	0.0004	0.004
	rs12709891	*GIPR*	A	C	-0.008	0.003
Systolic blood pressure[b]	rs55936433	*GIP*	C	A	0.010	0.002
	rs58274617	*GIP*	C	G	0.012	0.002

[a]Missense variant, only used in sensitivity analysis. [b]Co-localisation only at *GIP* gene. [c]Co-localisation only at *GIPR* gene.

ESM Table 4. Mendelian randomisation (MR) odds ratios (OR) or estimates (in standard deviation units) and their 95% confidence intervals (CI) per halving the odds of genetically proxied type 2 diabetes liability. SNP: single-nucleotide polymorphism.

**Outcome**		**SNPs**	**rsID**	**OR (95% CI)**	**p for difference[a]**
Heart failure	*GIP* and *GIPR* pooled	4		1.05 (0.65; 1.70)	
	GIP gene	2	rs55936433; rs58274617	0.71 (0.49; 1.01)	
	GIPR gene	2	rs55669001; rs12709891	1.47 (0.89; 2.43)	
	Glycaemic control generally	896		0.92 (0.90; 0.93)	0.70
				**Estimate (95% CI)**	
Body mass index	*GIP* and *GIPR* pooled	3		-0.16 (-0.30; -0.02)	
	GIP gene	2	rs55936433; rs58274617	-0.17 (-0.41; 0.08)	
	GIPR gene	1	rs12709891	-0.16 (-0.24; -0.08)	
	Glycaemic control generally	905		-0.06 (-0.07; -0.05)	0.07
C-reactive protein	*GIP* and *GIPR* pooled	4		-0.13 (-0.19; -0.08)	
	GIP gene	2	rs55936433; rs58274617	-0.17 (-0.24; -0.09)	
	GIPR gene	2	rs9749185; rs12709891	-0.11 (-0.20; -0.02)	
	Glycaemic control generally	906		-0.05 (-0.06; -0.03)	<0.001
High-density lipoprotein cholesterol	*GIP* and *GIPR* pooled	4		0.19 (0.14; 0.25)	
	GIP gene	2	rs55936433; rs58274617	0.19 (0.12; 0.26)	
	GIPR gene	2	rs55669001; rs12709891	0.20 (0.08; 0.31)	
	Glycaemic control generally	903		0.08 (0.06; 0.09)	<0.001
Triacylglycerol	*GIP* and *GIPR* pooled	4		-0.17 (-0.22; -0.12)	
	GIP gene	2	rs55936433; rs58274617	-0.19 (-0.26; -0.12)	
	GIPR gene	2	rs55669001; rs12709891	-0.14 (-0.24; -0.05)	
	Glycaemic control generally	903		-0.08 (-0.09; -0.06)	<0.001

SNPs = number of SNPs used; [a]p-value for testing the pooled MR estimate for *GIP* and *GIPR* variants being greater in magnitude than that for lower diabetes liability and improved glycaemic control generally.

ESM Table 5. Mendelian randomisation (MR) odds ratios (OR) or estimates (in standard deviation units) and their 95% confidence intervals (CI) per one unit (mmol/mol) decrease in genetically proxied HbA_{1c}. SNP: single-nucleotide polymorphism.

**Outcome**		**SNPs**	**rsID**	**OR (95% CI)**	**p for difference[a]**
Heart failure	*GIP* and *GIPR* pooled	4		1.09 (0.78; 1.52)	
	GIP gene	2	rs55936433; rs58274617	0.77 (0.55; 1.07)	
	GIPR gene	2	rs55669001; rs12709891	1.33 (1.11; 1.59)	
	Glycaemic control generally	896		0.96 (0.95; 0.96)	0.80
				**Estimate (95% CI)**	
Body mass index	*GIP* and *GIPR* pooled	3		-0.14 (-0.27; 0.00)	
	GIP gene	2	rs55936433; rs58274617	-0.12 (-0.34; 0.09)	
	GIPR gene	1	rs12709891	-0.17 (-0.25; -0.09)	
	Glycaemic control generally	905		-0.03 (-0.04; -0.02)	0.07
C-reactive protein	*GIP* and *GIPR* pooled	4		-0.09 (-0.15; -0.03)	
	GIP gene	2	rs55936433; rs58274617	-0.13 (-0.19; -0.07)	
	GIPR gene	2	rs9749185; rs12709891	-0.06 (-0.16; 0.03)	
	Glycaemic control generally	906		-0.03 (-0.04; -0.02)	0.02
High-density lipoprotein cholesterol	*GIP* and *GIPR* pooled	4		0.13 (0.06; 0.20)	
	GIP gene	2	rs55936433; rs58274617	0.16 (0.10; 0.21)	
	GIPR gene	2	rs55669001; rs12709891	0.11 (-0.03; 0.26)	
	Glycaemic control generally	903		0.04 (0.03; 0.05)	0.006
Triacylglycerol	*GIP* and *GIPR* pooled	4		-0.11 (-0.17; -0.04)	
	GIP gene	2	rs55936433; rs58274617	-0.15 (-0.21; -0.10)	
	GIPR gene	2	rs55669001; rs12709891	-0.08 (-0.19; 0.03)	
	Glycaemic control generally	903		-0.04 (-0.05; -0.03)	0.02

SNPs = number of SNPs used; [a]p-value for testing the pooled MR estimate for *GIP* and *GIPR* variants being greater in magnitude than that for lower diabetes liability and improved glycaemic control generally.

ESM Table 6. Mendelian randomisation (MR) odds ratios (OR) or estimates (in standard deviation units) and their 95% confidence intervals (CI) per halving the odds of genetically proxied type 2 diabetes liability, using missense variant rs2291725 in GIP or expression quantitative trait locus (eQTL) rs12709891 in GIPR.

**Outcome**		**rsID**	**OR (95% CI)**
Heart failure	*GIP* missense variant	rs2291725	0.67 (0.49; 0.92)
	GIPR eQTL	rs12709891	1.06 (0.75; 1.51)
			**Estimate (95% CI)**
Body mass index	*GIP* missense variant	rs2291725	-0.14 (-0.21; -0.07)
	GIPR eQTL	rs12709891	-0.16 (-0.24; -0.08)
C-reactive protein	*GIP* missense variant	rs2291725	-0.11 (-0.20; -0.01)
	GIPR eQTL	rs12709891	-0.16 (-0.27; -0.06)
High-density lipoprotein cholesterol	*GIP* missense variant	rs2291725	0.24 (0.15; 0.33)
	GIPR eQTL	rs12709891	0.27 (0.17; 0.37)
Triacylglycerol	*GIP* missense variant	rs2291725	-0.28 (-0.37; -0.19)
	GIPR eQTL	rs12709891	-0.20 (-0.30; -0.10)

ESM Table 7. Mendelian randomisation (MR) odds ratios (OR) or estimates (in standard deviation units) and their 95% confidence intervals (CI) per halving the odds of genetically proxied glycemic control for outcomes co-localising at only one locus. SNP: single-nucleotide polymorphism.

**Outcome**		**SNPs**	**rsID**	**OR (95% CI)**	**p for difference[a]**
Coronary artery disease	*GIP* gene	2	rs55936433; rs58274617	0.51 (0.37; 0.71)	
	Glycaemic control generally	896		0.87 (0.86; 0.89)	<0.001
				**Estimate (95% CI)**	
Alanine aminotransferase	*GIP* gene	2	rs55936433; rs58274617	-0.13 (-0.20; -0.07)	
	Glycaemic control generally	903		-0.07 (-0.08; -0.06)	0.03
Low-density lipoprotein cholesterol	*GIPR* gene	2	rs55669001; rs12709891	0.06 (-0.09; 0.21)	
	Glycaemic control generally	903		0.03 (0.02; 0.04)	0.65
Systolic blood pressure	*GIP* gene	2	rs55936433; rs58274617	-0.18 (-0.25; -0.12)	
	Glycaemic control generally	880		-0.03 (-0.04; -0.03)	<0.001

SNPs = number of SNPs used; [a]p-value for testing the locus specific MR estimate being greater than the MR estimate for lower diabetes liability and improved glycaemic control generally.

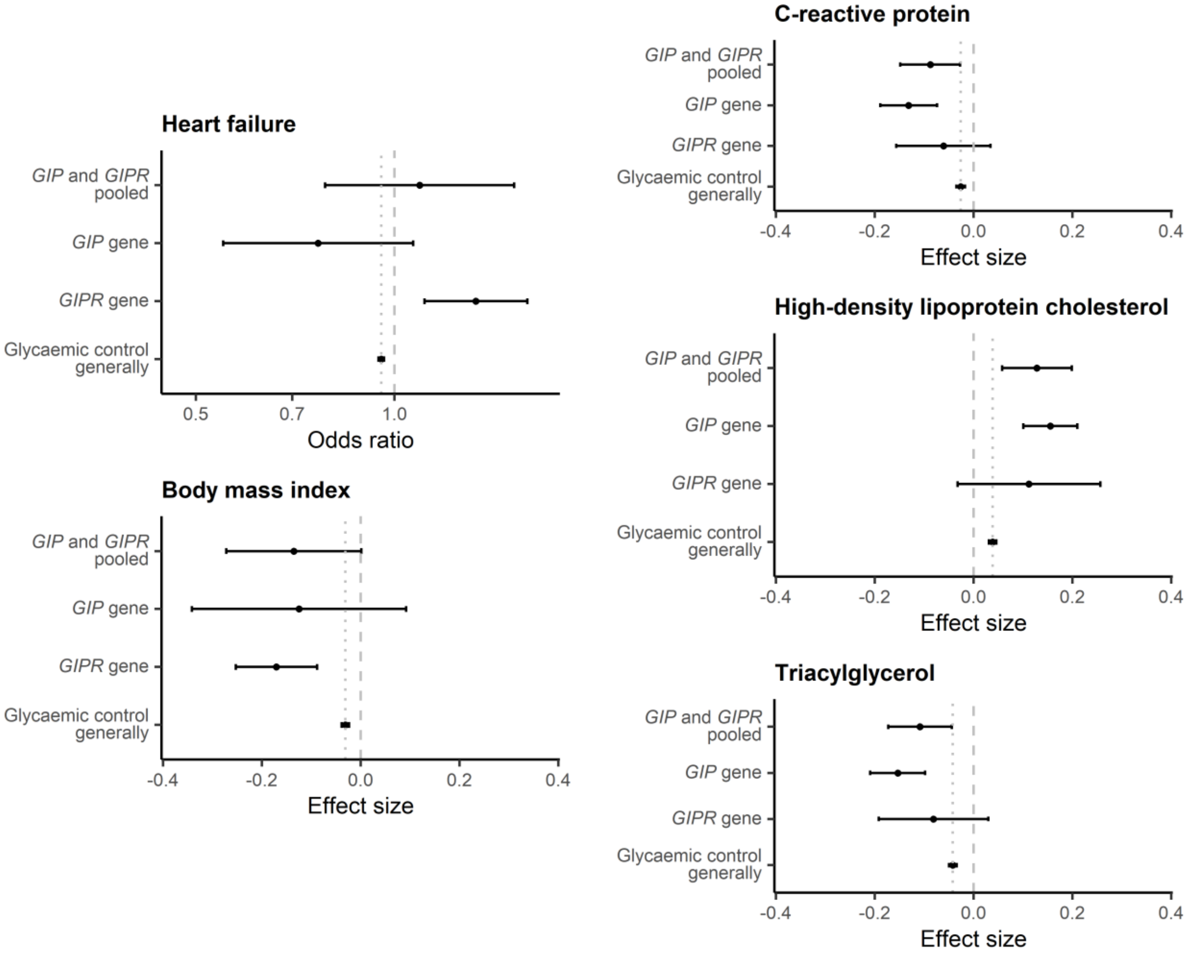

ESM Fig. 1. Odds ratios (for risk of heart failure) or estimate (Mendelian randomisation beta coefficients for body mass index, C-reactive protein, high-density lipoprotein cholesterol and triacylglycerol, all in standard deviation units) and their 95% confidence intervals per one unit (mmol/mol) decrease in genetically proxied HbA_{1c} levels. The dashed vertical line represents the null, and the dotted vertical line represents the estimates for glycaemic control generally.

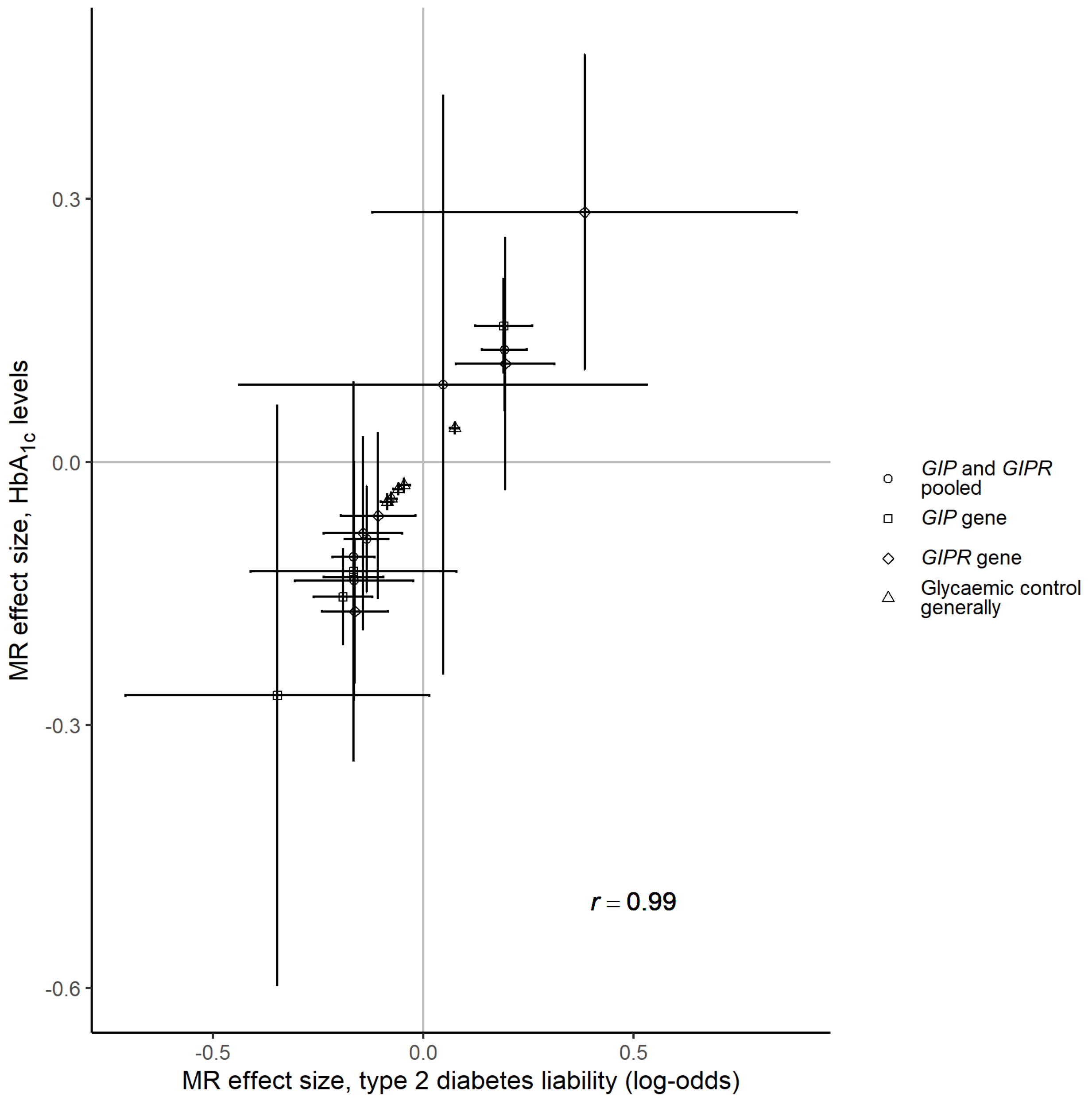

ESM Fig. 2. Comparison of the Mendelian randomisation (MR) estimates between variant-exposure association estimates based on type 2 diabetes liability (log-odds scale, x-axis) and HbA_{1c} levels (y-axis).

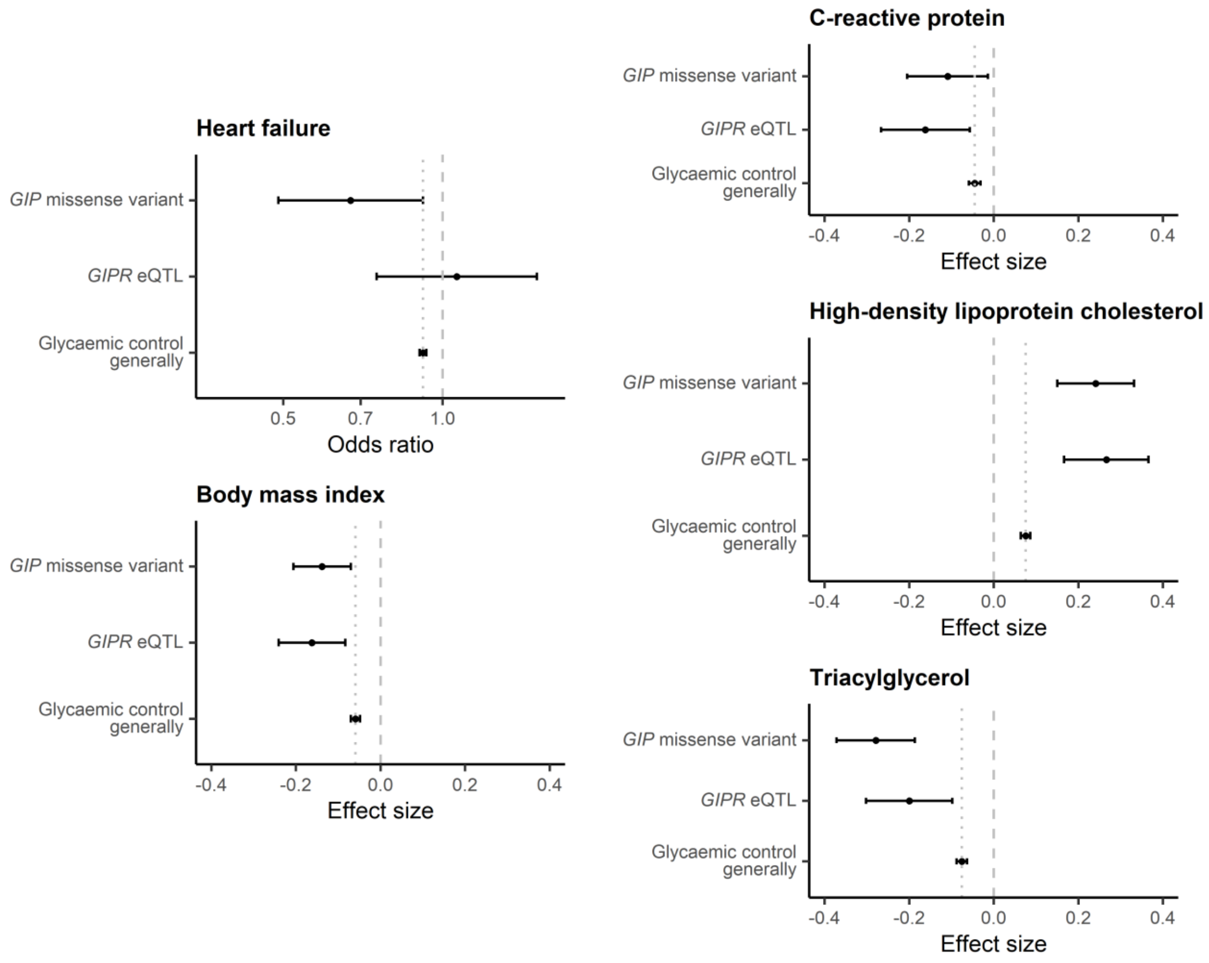

ESM Fig. 3. Mendelian randomisation odds ratios (for risk of heart failure) or effect size estimates (beta coefficients for body mass index, C-reactive protein, high-density lipoprotein cholesterol and triacylglycerol levels, all in standard deviation units) and their 95% confidence intervals per halving the odds of genetically proxied type 2 diabetes liability, using missense variant rs2291725 in *GIP* or expression quantitative trait locus (eQTL) rs12709891 in *GIPR*. The dashed vertical line represents the null, and the dotted vertical line represents the estimates for glycaemic control generally.

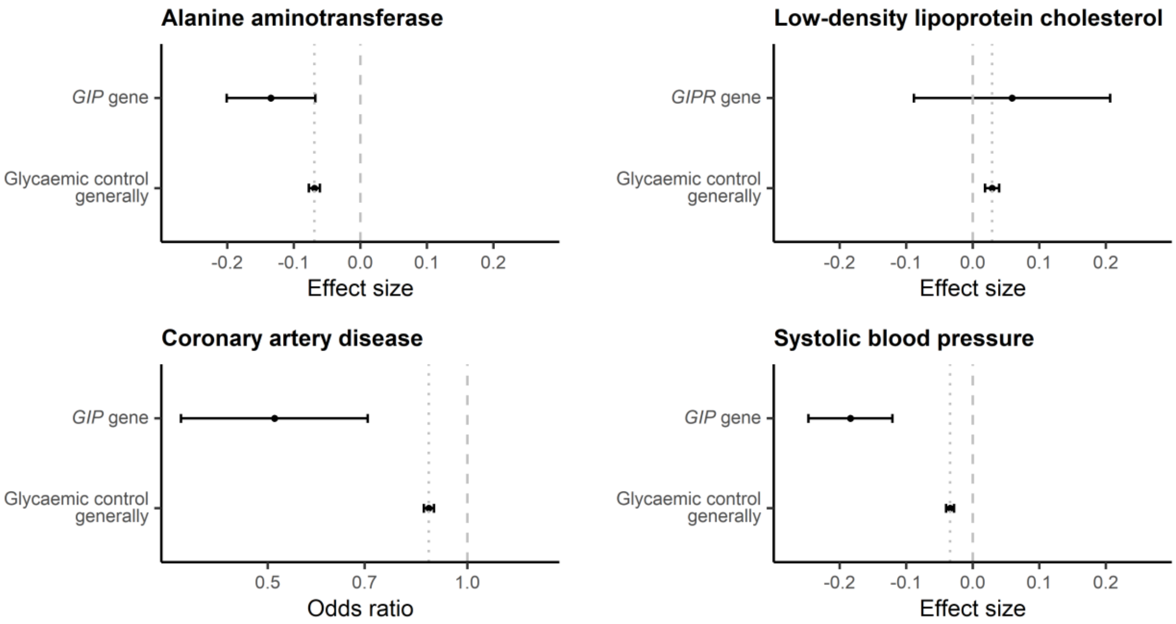

ESM Fig. 4. Mendelian randomisation odds ratios (for risk of coronary artery disease) or beta coefficients (for alanine aminotransferase, low-density lipoprotein cholesterol and systolic blood pressure, all in standard deviation units) and their 95% confidence intervals per halving the odds of genetically proxied type 2 diabetes liability. The dashed vertical line represents the null association, and the dotted vertical line represents the association of type 2 diabetes liability generally.

References

1. Mahajan A, Taliun D, Thurner M, et al (2018) Fine-mapping type 2 diabetes loci to single-variant resolution using high-density imputation and islet-specific epigenome maps. Nature Genetics 50(11):1505–1513. https://doi.org/10.1038/s41588-018-0241-6

2. Vujkovic M, Keaton JM, Lynch JA, et al (2020) Discovery of 318 new risk loci for type 2 diabetes and related vascular outcomes among 1.4 million participants in a multi-ancestry meta-analysis. Nature Genetics 52:680–691. https://doi.org/10.1038/s41588-020-0637-y

3. The Neale Lab (2018) Updated GWAS Analysis of the UK Biobank. http://www.nealelab.is/uk-biobank. Accessed 3 Feb 2021

4. Wuttke M, Li Y, Li M, et al (2019) A catalog of genetic loci associated with kidney function from analyses of a million individuals. Nature genetics 51(6):957. https://doi.org/10.1038/s41588-019-0407-x

5. Nikpay M, Goel A, Won H-H, et al (2015) A comprehensive 1000 Genomes–based genome-wide association meta-analysis of coronary artery disease. Nature genetics 47(10):1121. https://doi.org/10.1038/ng.3396

6. Shah S, Henry A, Roselli C, et al (2020) Genome-wide association and Mendelian randomisation analysis provide insights into the pathogenesis of heart failure. Nature communications 11(1):1–12. https://doi.org/10.1038/s41467-019-13690-5

7. Malik R, Chauhan G, Traylor M, et al (2018) Multiancestry genome-wide association study of 520,000 subjects identifies 32 loci associated with stroke and stroke subtypes. Nature Genetics 50:524–537. https://doi.org/10.1038/s41588-018-0058-3

8. Pulit SL, Stoneman C, Morris AP, et al (2018) Meta-analysis of genome-wide association studies for body fat distribution in 694 649 individuals of European ancestry. Human Molecular Genetics 28(1):166–174. https://doi.org/10.1093/hmg/ddy327

9. Evangelou E, Warren HR, Mosen-Ansorena D, et al (2018) Genetic analysis of over 1 million people identifies 535 new loci associated with blood pressure traits. Nature Genetics 50(10):1412–1425. https://doi.org/10.1038/s41588-018-0205-x

10. Bailey CJ (2020) GIP analogues and the treatment of obesity-diabetes. Peptides 125:170202. https://doi.org/10.1016/j.peptides.2019.170202

11. Giambartolomei C, Vukcevic D, Schadt EE, et al (2014) Bayesian Test for Colocalisation between Pairs of Genetic Association Studies Using Summary Statistics. PLOS Genetics 10(5):1–15. https://doi.org/10.1371/journal.pgen.1004383

12. Burgess S, Butterworth A, Thompson SG (2013) Mendelian Randomization Analysis With Multiple Genetic Variants Using Summarized Data. Genetic Epidemiology 37(7):658–665. https://doi.org/10.1002/gepi.21758

13. Lee SH, Goddard ME, Wray NR, Visscher PM (2012) A Better Coefficient of Determination for Genetic Profile Analysis. Genetic Epidemiology 36(3):214–224. https://doi.org/10.1002/gepi.21614

14. Staley JR, Blackshaw J, Kamat MA, et al (2016) PhenoScanner: a database of human genotype–phenotype associations. Bioinformatics 32(20):3207–3209. https://doi.org/10.1093/bioinformatics/btw373

15. Kamat MA, Blackshaw JA, Young R, et al (2019) PhenoScanner V2: an expanded tool for searching human genotype–phenotype associations. Bioinformatics 35(22):4851–4853. https://doi.org/10.1093/bioinformatics/btz469

16. GTEx Consortium (2017) Genetic effects on gene expression across human tissues. Nature 550(7675):204–213. https://doi.org/10.1038/nature24277

17. Wasserstein RL, Schirm AL, Lazar NA (2019) Moving to a World Beyond "p < 0.05." The American Statistician 73(sup1):1–19. https://doi.org/10.1080/00031305.2019.1583913
